# Selection of suitable reference genes for qPCR normalization in different developmental stages of *Oenanthe javanica*

**DOI:** 10.3389/fpls.2023.1287589

**Published:** 2023-12-27

**Authors:** Kai Feng, Zhi-Yuan Yang, Ya-Jie Yan, Nan Sun, Zi-Qi Zhou, Jia-Lu Liu, Shu-Ping Zhao, Peng Wu, Liang-Jun Li

**Affiliations:** ^1^ College of Horticulture and Landscape Architecture, Yangzhou University, Yangzhou, China; ^2^ Joint International Research Laboratory of Agriculture and Agri−Product Safety of Ministry of Education of China, Yangzhou University, Yangzhou, China

**Keywords:** water dropwort, development stages, reference genes, RT-qPCR, growth stages

## Abstract

Gene expression analysis is widely used to unravel molecular regulatory mechanisms and identify key genes in plants. Appropriate reference gene is an important prerequisite to ensure the accuracy and reliability of qPCR analysis results. Water dropwort is a plant of the Oenanthe genus in the Apiaceae family, which has high economic benefits. However, the underlying molecular regulatory mechanisms in the growth and development of water dropwort have not been fully understood and the appropriate reference genes in different developmental stages of water dropwort not yet reported. In this study, 10 candidate reference genes (*ACTIN, PP2A, SAND, EF-1α, GAPDH, UBQ, MIP, TBP, RPS-18, eIF-4α*) were identified and cloned from *Oenanthe javanica*. The qPCR primers of candidate reference genes were designed and verified. Four statistical algorithms, geNorm, NormFinder, BestKeeper and RefFinder were used to evaluate the expression stability of 10 candidate reference genes in different developmental stages of water dropwort. The results showed that *TBP* and *UBQ* were the most stable genes in different developmental stages of water dropwort, while *GAPDH* was the most unstable gene. The normalization of *EXP1* genes at different developmental stages further confirmed the reliability of internal reference genes. The results of this study provide a theoretical basis for selecting appropriate internal reference genes in different developmental stages of water dropwort. This study also provides technical support and reliable basis for the expression analysis of key genes in different developmental stages of water dropwort.

## 1 Introduction

Water dropwort [*Oenanthe javanica* (Blume) DC.] is a plant of the Oenanthe genus in the Apiaceae family (Lu and Li, 2019). Water dropwort is widely cultivated in the countries of East Asian and is a popular vegetable in China because of its special aroma and crisp taste (Ai et al., 2016; Feng et al., 2022). Water dropwort has both medicinal and edible value, which contains rich nutrients such as protein, dietary fiber, vitamins (Feng et al., 2018; Feng et al., 2023a; Feng et al., 2023c). Water dropwort contains hyperoside, persicarin and isorhamnetin with pharmacological activity (Jiang et al., 2014), which has the functions of liver antihypertensive (Chen et al., 1999), antithrombotic (Ku et al., 2013), sedative and anticancer (Kim et al., 2011). However, different plants usually contain their own stable genes, which may be show various expression stabilities under different developmental stages (Czechowski et al., 2005). During plant development, the physiological morphological characteristics of water dropwort changed significantly, which affected its edible quality and nutritional quality. The developmental changes of leaves and stems of water dropwort are controlled by complex regulatory mechanisms. Therefore, it is very important to study the gene function and gene expression pattern related to the development stage of water dropwort and the suitable reference gene is necessary for gene expression analysis fin water dropwort.

Gene expression analysis is a common method to understand molecular regulation mechanism and identify key genes (Bustin et al., 2005; Silva et al., 2019). Real-time quantitative polymerase chain reaction (RT-qPCR) is widely used in agronomy, genetics gene expression detection and molecular technology quantification on account of its high sensitivity, high accuracy, high specificity, low cost and easy operation (Gachon et al., 2004; Bustin et al., 2005; Derveaux et al., 2010; Miao et al., 2019). However, RNA stability, purity, cDNA quality, reverse transcription efficiency and other factors greatly affect the accuracy and reliability of RT-qPCR results, and internal reference genes are also important factors (Jo et al., 2002). Some genes play a house-keeping role in basic cellular biological processes, for instance the maintenance of cell structure and primary metabolism, and are called house-keeping genes, also known as internal reference genes (Gutierrez et al., 2008). The optimum reference gene should be stably expressed in all tissues, developmental stages and under experimental conditions (Keertan et al., 2004). At present, the expression of no internal reference gene is always constant (Volkov et al., 2003). Therefore, in different species, it is essential to select appropriate internal reference genes to ensure the accuracy and reliability of the test results. In general, gene expression can be standardized or quantified by selecting one or more stable internal reference genes.

At present, a variety of reference genes with stable expression and reliability in plants have been reported. However, the stability of reference genes in different plants are not consistent. The expression of reference genes that are stable in one plant may be unstable in another plant (Tian et al., 2015). For example, *RuEEF1A* and *Ru18S* are the most stable genes in raspberries and blackberries (Wu et al., 2021). The most stable genes in carrots were *ACTIN* and *TUB* (Tian et al., 2015). At the same time, the stability of reference genes were different in various growth environment and growth period. The expression levels of *TBP1* and *EIF4A1* were the most stable reference genes in *Gleditsia microphylla* under cold, hot and dry growth conditions and hormone treatment (Yang et al., 2022). *elF-4α* and *ACT1* were the most stable internal reference genes at different developmental stages of rice seeds (Li et al., 2009). In celery, *TUB-A*, *TUB-B* and *UBC* were the most stable internal reference genes at different developmental stages of leaves and petioles (Li et al., 2016). Previous studies showed that *ACT7* and *PP2A* were the most stable internal reference genes in water dropwort under abiotic stress (Jiang et al., 2014). However, the reference genes in different developmental stages of water dropwort not yet reported. Therefore, in order to analyze gene expression in water dropwort more accurately and reliably, reference genes in different developmental stages of water dropwort were screened in this study.

Based on previous studies, 10 candidate reference genes (*ACTIN, PP2A, SAND, EF-1α, GAPDH, UBQ, MIP, TBP, RPS-18, eIF-4α*) were selected for stability analysis (Libault et al., 2008; Wan et al., 2011; Galli et al., 2013; Kozera and Rapacz, 2013; Monteiro et al., 2013). Detection of expression levels of candidate reference genes in different developmental stages of water dropwort using RT-qPCR method. Four different algorithms geNorm (Jo et al., 2002), NormFinder (Lund et al., 2011), BestKeeper (Claus et al., 2004) and RefFinder (Xie et al., 2012) were used to evaluate the stability of candidate reference genes. Meanwhile, in order to verify the reliability of the selected internal reference genes, the expression level of *EXP1* gene, a major factor regulating cell wall extension and organ expansion in aquatic plants, was evaluated by using candidate internal reference genes (Liu et al., 2022; Feng et al., 2023b). The purpose of this study was to screen the internal reference genes with stable expression in different developmental stages of water dropwort. These results will provide technical support and reliable basis for the expression analysis of key genes in different developmental stages of water dropwort.

## 2 Materials and methods

### 2.1 Plant materials and experimental treatments

**Figure 1:**
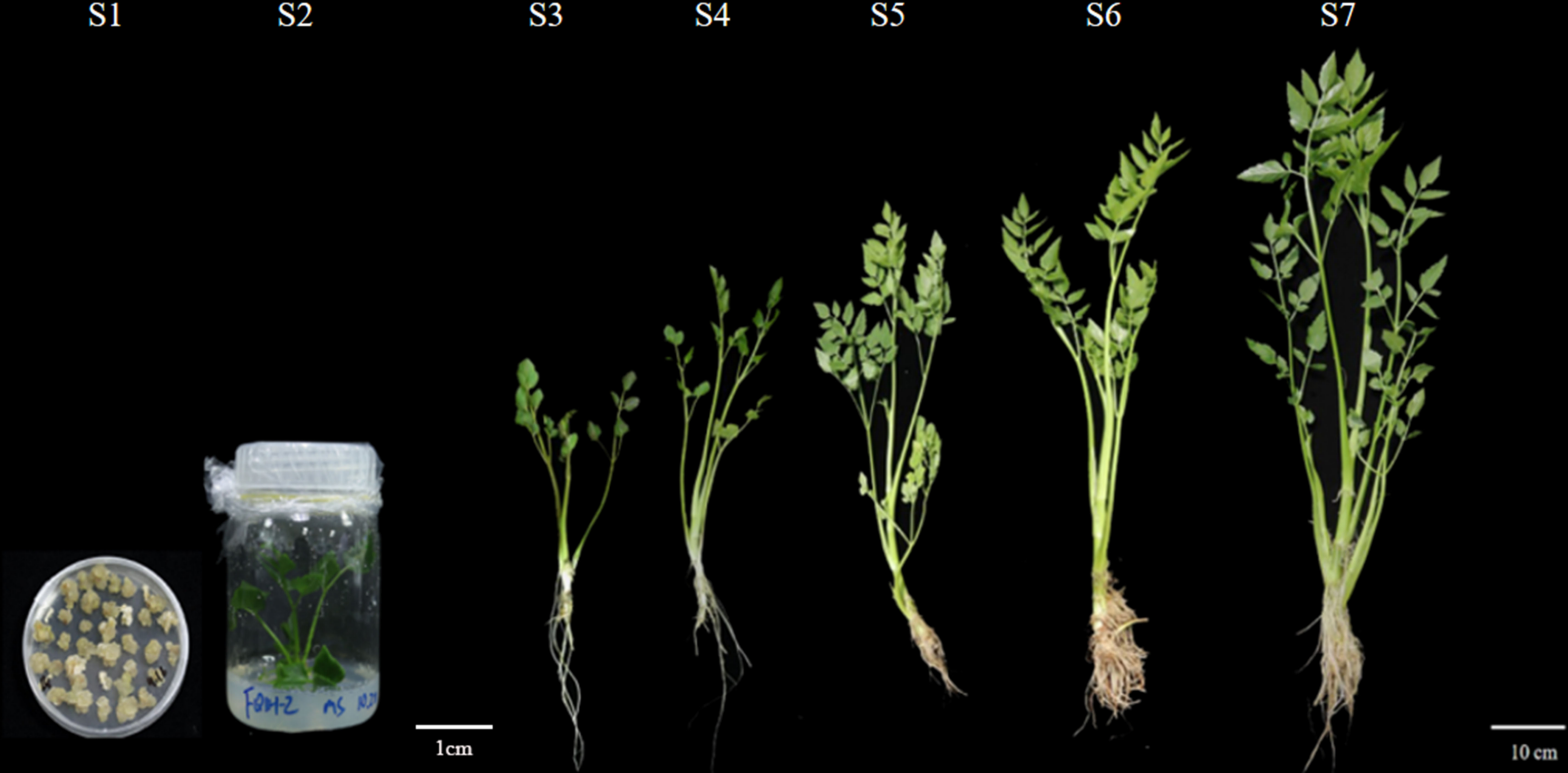
Growth status of water dropwort at seven developmental stages. Stage 1, Water dropwort callus; Stage 2, Water dropwort regenerate seedlings; Stage 3, Growth and development for 30 days; Stage 4, Growth and development for 45 days; Stage 5, Growth and development for 60 days; Stage 6, Growth and development for 75 days; Stage 7,Growth and development for 90 days.

The callus and regenerates seedlings of water dropwort were cultured in a controlled-environment growth chamber at Yangzhou University, Yangzhou, China (32°23’ N, 119°25’ E). All plants were grown at 25 °C under 400 μmol m^-2^s^-1^ light intensity for 16 h and at 16 °C darkness for 8 h, the relative humidity is controlled at 60~ 70%. Seven stages of different growth and development of water dropwort were evaluated. The first stage is the callus, and the second stage is the regenerates seedlings obtained from callus. The third, fourth, fifth, sixth and seventh stages of water dropwort were 30 d, 45 d, 60 d, 75 d and 90 d after planted, respectively (**Figure 1**). Three biological replicate samples of the samples of water dropwort were collected and immediately frozen with liquid nitrogen and stored in a refrigerator at -80°C for future use.

### 2.2 RNA extraction and cDNA reverse transcription

RNA Simple Total RNA Kit (Tiangen, Beijing, China) was used to extract total RNA from different growth and development stages of water dropwort. NanoDrop 2000 (Thermo, Waltham, USA) were used to detected RNA concentration and purity. According to the instructions, the total RNA of each sample was reverse-transcribed into cDNA using the HiScript III 1st Strand cDNA Synthesis Kit (Vazyme, Nanjing, China). The obtained cDNA was diluted with ddH_2_O and stored in the refrigerator at -20°C for subsequent RT-qPCR experiments.

### 2.3 Selection of candidate reference genes and primers design

In this study, 10 candidate reference genes (*ACTIN*, *PP2A*, *SAND*, *EF-1α*, *GAPDH*, *UBQ*, *MIP*, *TBP*, *RPS-18*, and *eIF-4α*) were selected for qPCR analysis based on previous researches about selection of suitable reference genes in other species (Libault et al., 2008; Wan et al., 2011; Galli et al., 2013; Kozera and Rapacz, 2013; Monteiro et al., 2013; Li et al., 2016). The sequence of Arabidopsis homolog locus were download from TAIR database (http://www.arabidopsis.org), which were used as query sequences to search homologous genes in *Oenanthe javanica*. Based on the transcriptome and genome sequencing data established by our research group, the above 10 candidate reference genes were further confirmed and cloned. Primer premier 6.0 software was used to design primers of candidate reference genes. The primer design criteria were as follows: GC content 45–65%, optimal Tm 55-65°C, primer length 15–25 bp and amplicon length 110–220 bp. The specificity of primers was identified by BLASTed in the database of water dropwort. The primers were synthesized by Shanghai Shenggong Bioengineering. Primers were screened by PCR and agarose gel electrophoresis. The primers with clear bands, good specificity and no primer dimer were selected for further qRT-PCR assay (Yang et al., 2022). The final primer with a single peak melting curve should be selected (Feng et al., 2019).

### 2.4 Quantitative RT-PCR analysis

qRT-PCR was performed using CFX96 Real-time PCR system (Bio-Rad). Each reaction contained 0.4 μL of each forward and reverse primer, 2 μL of template cDNA, 10μL ChamQ Universal SYBR qPCR Master Mix (Vazyme, Nanjing, China), and make up to 20μL with ddH_2_O. The qRT-PCR reaction conditions were as follows: 95 °C for 2 min, then 95 °C 5 s, 60 °C 30 s in a group, 40 cycles. The melt curve analysis at 55-95°C using default parameters at the end of each experiment. Three technical repeats and three biological repeats were performed for each candidate reference gene at each stage of development (**Supplementary Table S1**). The standard curve qPCR was performed using The 10-fold, 10^2^-fold, 10^3^-fold, 10^4^-fold, 10^5^-fold, 10^6^-fold, 10^7^-fold and 10^8^-fold diluted cDNA as the template. The standard curve was established with Ct value as the ordinate and logarithm of dilution multiple as the abscate. The amplification efficiency (E) and correlation coefficient (R^2^) were calculated by the established standard curve, and the amplification efficiency was calculated as % E =(10^(−1/slope)^ -1)×100% (Radonic et al., 2004) (**Table 1**).

**Table 1:** Descriptions of candidate reference genes in *O. javanica*.

Gene symbol	Gene name	Arabidopsis homolog locus	Primer sequence forward/reverse	Amplicon length (bp)	Amplification efficiency (E %)	Correlation coefficient(R^2^)
*PP2A*	Protein phosphatase 2A gene	AT4G15415	CAGATAGGTCGCTGCCTCAACA /GGACAGCTTGGTTCCAGTGACT	1569	91.9	0.991
*ACT7*	Actin7 gene	AT5G09810	ACCACTGCTGAACGGGAAATCG/GCTGGAACAGGACTTCTGGACC	1134	100.2	0.997
*GAPDH*	Glyceraldehyde-3-phosphate dehydrogenase gene	AT1G42970	GAGACTTGAGGAGAGCCAGAGC/GGCACCCGTAGAGCAATACCAT	1344	110	0.997
*EF-1α*	Elongation factor -1α gene	AT1G07940	TGGTGATGCTGGGATGGTCAAG /CCAACGGCAACTGTCTGTCTCA	1350	106.5	0.997
*eIF-4α*	Eukaryotic translation initiation factor 4α-1 gene	AT3G13920	GGTTGGTACTCCTGGTCGTGTA /GGAGGCATGGTGGCAGAGAA	1260	109.2	0.999
*TBP*	TATA-box binding protein gene	AT1G55520	AAGGTAGCCAACCAGTGGATCT /GATTCGCATAATGACAGCAGCA	603	101.8	0.992
*SAND*	SAND family protein gene	AT2G28390	AGATGACGACTCCACTGACCAA /AAGACGAATCGGAAGGCAATGT	1824	93.3	0.992
*UBQ*	UBQ family protein gene	AT1G55060	CCTGGAGGTGGAGAGTTCGGAT /CACGGAGACGCAACACCAAGT	693	108.2	0.992
*MIP*	MIP family protein gene	AT1G01620	GGCGGTGGTGCTAATGTTGTG /ATAGGAACGTGCGAGTCTCTGG	861	95.5	0.997
*RPS18*	RPS family protein gene	AT1G22780	AGAGATGATCTTGAGCGCCTGA /CGTCCCGTAGTCTTGGTGTGTT	459	107.4	0.999

### 2.5 Data analysis

In this study, the expression data were used to selection the appropriate reference gene. According to previous research, the original Ct value (Cq value in this paper) was analyzed by using statistical software of four different algorithms, so as to rank the stability of different internal reference genes and determine the stable internal reference genes (Feng et al., 2019; Liu et al., 2022). The RT-qPCR data of different developmental stages of water dropwort were sorted and summarized by excel 2022 software. The stability analysis software geNorm (Jo et al., 2002), NormFinder (Lund et al., 2011) and BestKeeper (Claus et al., 2004) were used to evaluate and rank the stability of ten candidate reference genes in different developmental stages of the water dropwort (each sample included three biological repeats and technical repeats).

(1) geNorm. When the geNorm algorithm is used to analyze the gene expression stability (M value) (Jo et al., 2002), the original Ct value needs to be converted to 2 ^− ΔCt^values (delta Ct = original Ct value − the lowest Ct value in each group). The stability of the gene was evaluated according to the mean variation of M value. When the M value is greater than 1.5, this gene is not suitable as an internal reference gene. If the M value is less than 1.5, the smaller the M value, the higher the stability. The V value of pairing variation analysis in geNorm algorithm can help to identify optimal number of candidate reference genes. The default V value threshold is 0.15. When Vn/n +1 < 0.15, the optimal number of internal reference genes is n; otherwise, the optimal number of internal reference genes is n +1.

(2) NormFinder. NormFinder algorithm calculated the stability value of the internal reference gene expression by 2 ^− ΔCt^ values. The lower the stability value, the higher the stability of the selected gene, and the more suitable to be selected as the internal reference gene (Lund et al., 2011).

(3) BestKeeper. The BestKeeper algorithm calculated standard deviation (SD) and coefficient of variation (CV) according to the initial Ct values of candidate reference genes to evaluate their expression stability. When SD-values is greater than 1, this gene cannot be used as internal reference gene. The smaller the SD and CV-values, the more stable the gene expression (Claus et al., 2004).

(4) RefFinder. The RefFinder algorithm can comprehensively evaluate and rank the expression stability of all candidate reference genes according to the analysis results of the above three kinds of software, so as to determine the most stable gene expression (Xie et al., 2012).

### 2.6 Validation of reference genes

The relative expression levels of *EXP1*, a major factor regulating cell wall extension and plant organ expansion in different developmental stages were analyzed using single stably expressed gene and single unstable expressed gene according to the 2 ^− ΔΔct^ method (Schmittgen and Livak, 2008) to verify the stability and reliability of the selected reference genes.

## 3 Results

### 3.1 Amplification efficiency and primer specificity analysis

In this study, 10 genes (*ACTIN, PP2A, SAND, EF-1α, GAPDH, UBQ, MIP, TBP, RPS-18, eIF-4α)* were selected as candidate reference genes. The specificity and amplification efficiency of the primers were verified by PCR amplification and RT-qPCR melting curve. The candidate primers fully amplified all 10 internal reference genes, and the product length was 100-250bp (**Figure S1**). RT-qPCR results showed that the melting curves of all candidate reference genes were unimodal, which indicate all primers had good specificity (**Supplementary Figure S2**). In the meantime, the standard curves of 10 candidate reference genes were constructed (**Supplementary Figure S3**). The results showed that the amplification efficiency (E) of 10 candidate reference genes ranged from 91.9 to 110% and the correlation coefficient (R^2^) was greater than 0.99 (**Table 1**). These results indicated that the amplification efficiency and primer specificity of each candidate reference gene met the criterion of qRT-PCR.

### 3.2 Analysis of Cq values of candidate reference genes

**Figure 2:**
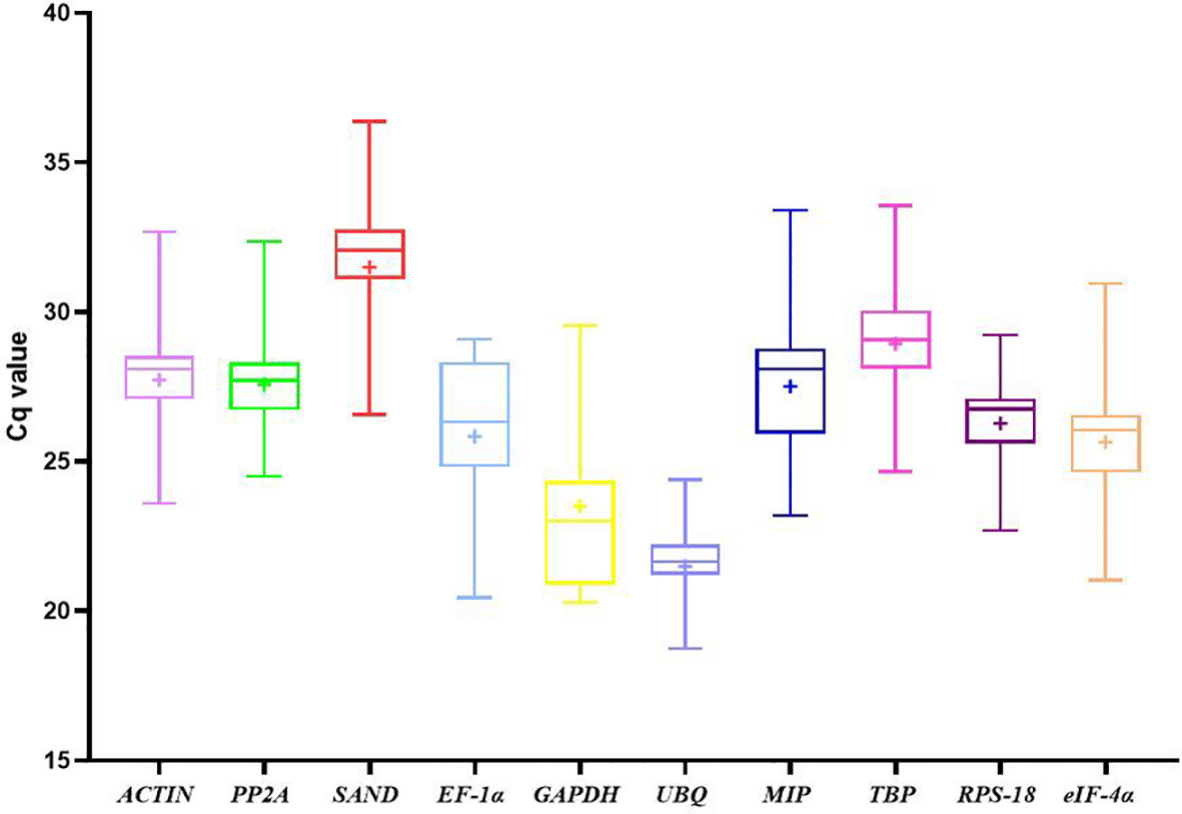
Cq values of candidate reference genes in all samples of water dropwort. The line across the box depicts median. The inside box depicts mean. The outside box is determined by the 25th and 75th percentiles. The whiskers are determined by the 5th and 95th percentiles.

**Figure 3:**
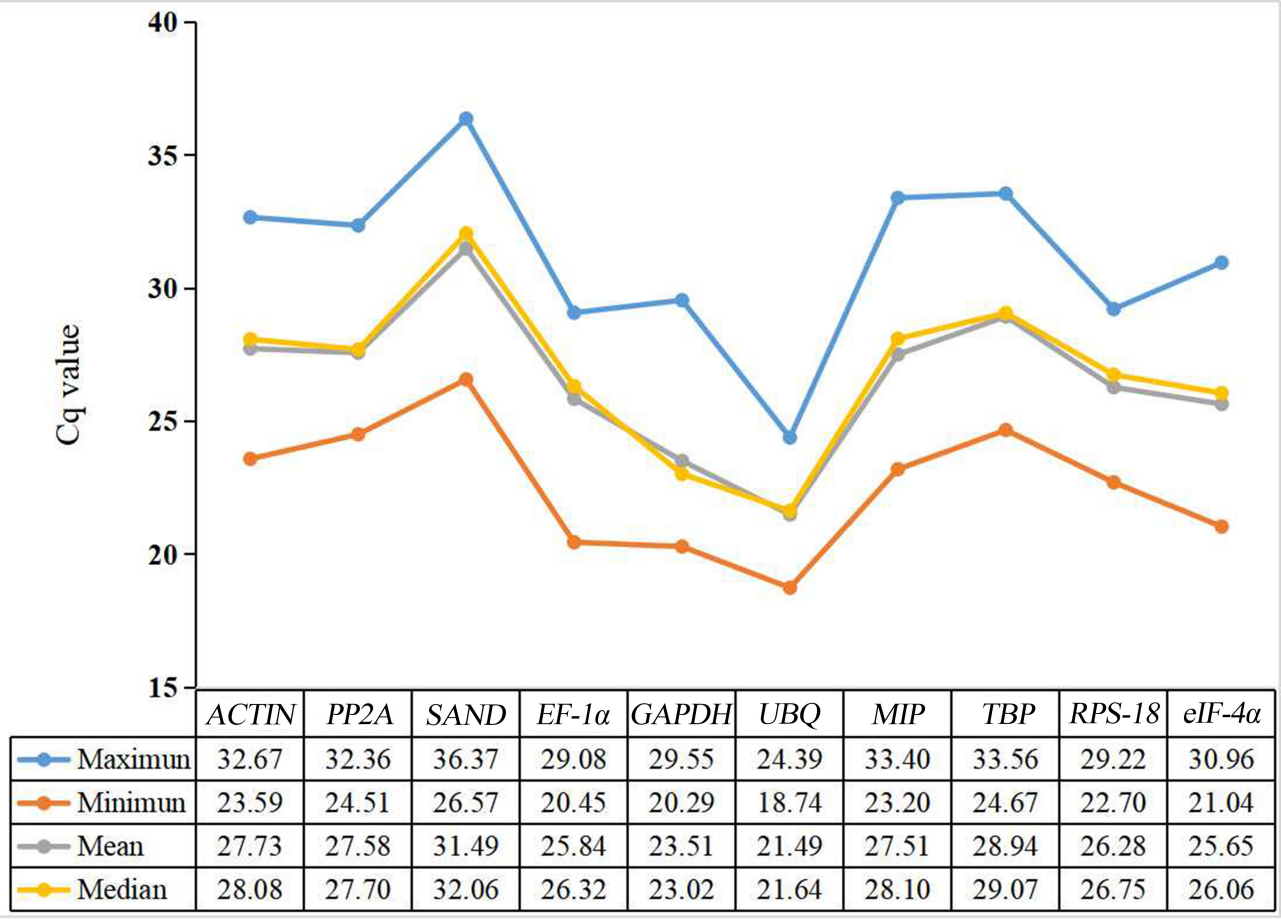
Statistic analysis (maximum, minimum, mean, and median) of Cq values of ten candidate reference genes in all samples.

Cq values were used to evaluate gene expression levels. There were significant differences in the range of Cq values of 10 candidate reference genes in different developmental stages of water dropwort (**Figure 2**). The smaller the Cq value, the higher the expression level of this gene. On the contrary, the expression level of this gene is lower. However, too high or too low Cq value is not conducive to subsequent target gene analysis. Generally, Cq value between 15-35 is considered appropriate and valid data (Czechowski et al., 2005; Derveaux et al., 2010). Except that the highest Cq value of *SAND* is 36.37, the Cq values of all candidate reference genes ranged from 18 to 35 (**Figure 3**). Among all candidate reference genes, *UBQ* gene had the highest average expression level, and the average Cq value was 21.49. The mean expression level of *SAND* gene was the lowest and the average Cq value was 31.49. The Cq value of *UBQ* gene was 18.74-24.39. The expression level of *UBQ* gene changed the least among all candidate reference genes. The expression level of *MIP* gene changed the most and its Cq value was 23.20-33.40. These results indicated that there were significant differences in the expression levels of candidate reference genes at different developmental stages. Their expression patterns were not completely stable across all samples. Therefore, four statistical algorithms were further used to evaluate the expression stability of 10 candidate reference genes at different development stages of water dropwort.

### 3.3 Stability analysis of candidate reference genes

Four different statistical algorithms were used to analyze the expression stability of 10 candidate reference genes in callus, regenerates seedlings, plant growth at 30 d, 45 d, 60 d, 75 d and 90 d after planted. Since each gene in a single period has only one expression value, its expression value is not enough to prove the stability of the gene in that period. Therefore, in order to make the experimental results more accurately and reliably, we combined all samples to evaluate the stability of 10 candidate reference genes in different developmental stages of water dropwort.

#### 3.3.1 geNorm analysis

**Figure 4:**
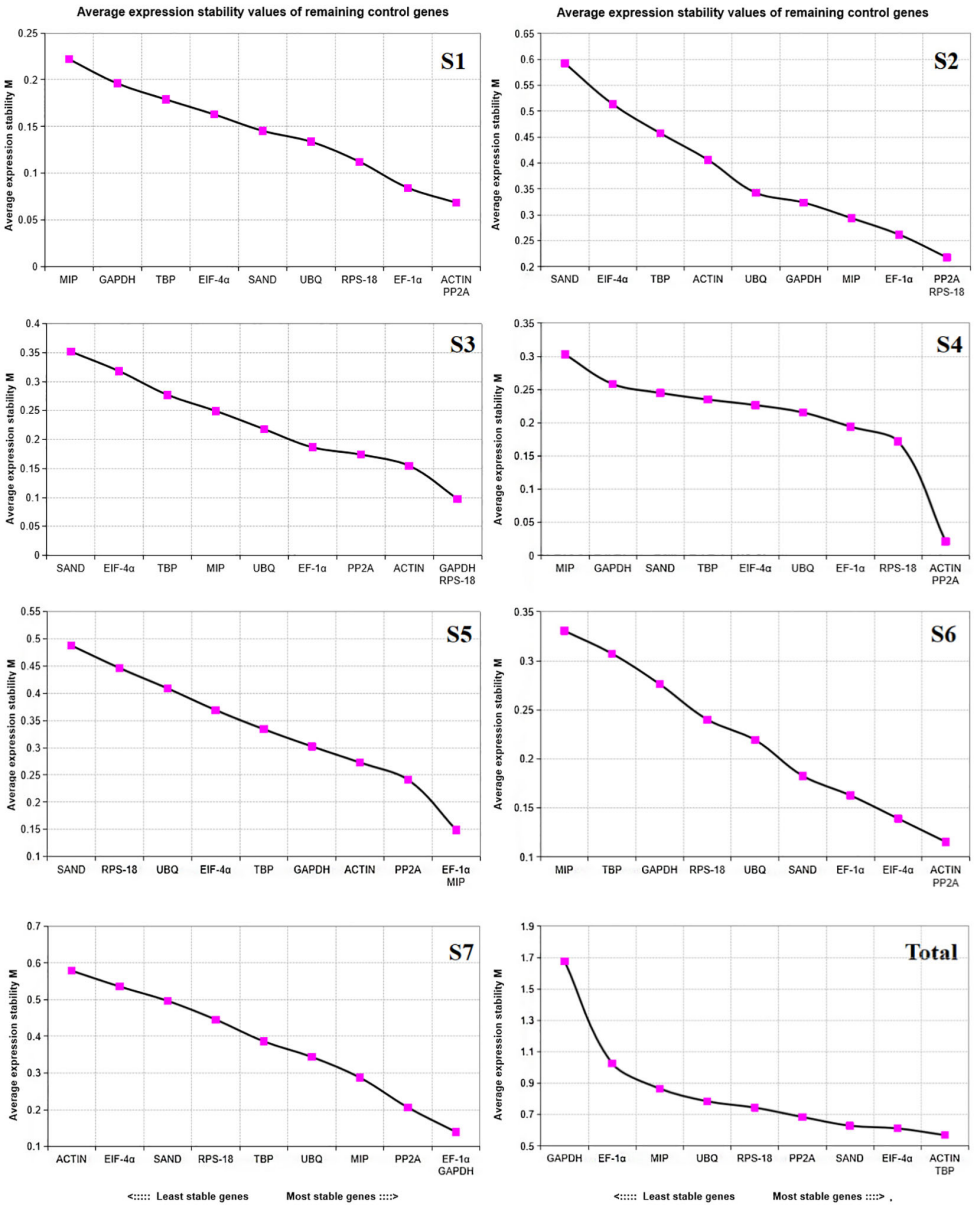
Measurement(M) of expression stability of 10 candidate reference genes in samples at different developmental stages using geNorm. The most unstable genes are on the left and the most stable genes are on the right. S1-S7: The seven stages of water dropwort development. Total represents samples from the above seven experimental conditions.

geNorm software sorted the stability of candidate reference genes by calculating their M values. The lower the M value, the more stable the expression of reference genes (Jo et al., 2002). Candidate reference genes with M value higher than 1.5 are not suitable for selection as reference genes. In all samples, the M value of candidate reference genes was less than 1.5 (**Table 2**). As shown in **Figure 4**, the stable internal reference genes were different at different developmental stages, in all samples, *ACTIN* and *TBP* had the smallest S value of 0.569 in different developmental stages of water dropwort, showing good stability in each developmental stage, *eIF-4α* has an s value of 0.613, which is second only to these two genes in terms of stability. *GAPDH* had the highest S value and was the most unstable gene in different developmental stages. In summary, *ACTIN*+*TBP* showed the best stability and were the most stable reference gene in each stage, while *GAPDH* was the most unstable reference gene in each stage.

**Table 2:** The expression stability of candidate reference genes under different development stages of water dropwort calculated by geNorm, NormFinder, BestKeeper and RefFinder.

	Rank	geNorm		NormFinder		BestKeeper			RefFinder
		Gene	Stability	Gene	Stability	Gene	SD	CV	Gene
Total	1	*ACTIN*	0.569	*UBQ*	0.067	*UBQ*	0.95	4.42	*TBP*
	2	*TBP*	0.569	*TBP*	0.097	*RPS-18*	1.23	4.69	*UBQ*
	3	*eIF-4α*	0.613	*RPS-18*	0.231	*TBP*	1.40	4.83	*ACTIN*
	4	*SAND*	0.630	*PP2A*	0.241	*PP2A*	1.41	5.11	*RPS-18*
	5	*PP2A*	0.685	*ACTIN*	0.315	*ACTIN*	1.50	5.40	*PP2A*
	6	*RPS-18*	0.745	*eIF-4α*	0.44	*eIF-4α*	1.74	6.79	*eIF-4α*
	7	*UBQ*	0.785	*SAND*	0.578	*SAND*	1.78	5.66	*SAND*
	8	*MIP*	0.866	*MIP*	0.68	*MIP*	1.98	7.19	*MIP*
	9	*EF-1α*	1.026	*EF-1α*	1.204	*EF-1α*	2.13	8.25	*EF-1α*
	10	*GAPDH*	1.676	*GAPDH*	2.929	*GAPDH*	2.34	9.94	*GAPDH*

#### 3.3.2 NormFinder analysis

Similar to geNorm, NormFinder software evaluates the expression stability of each candidate internal reference gene according to its stability value (Lund et al., 2011). The lower the S value, the higher the stability (**Table 2**). As can be seen from **Table 2**, *UBQ* and *TBP* had the smallest S value (0.067 and 0.097, respectively), which were the most stable internal reference genes in different developmental stages of water dropwort, while *RPS-18* had an S value of 0.231, which was the third stable internal reference gene expressed at each stage. *GAPDH*, with s value of 2.929, was the most unstable reference gene in all stages. According to the analysis of NormFinder software, *UBQ*+*TBP* were selected as the reference gene in different developmental stages of water dropwort more appropriate.

#### 3.3.3 BestKeeper analysis

The BestKeeper software determined the correlation coefficient (R^2^), standard deviation (SD) and coefficient of variation (CV) according to the Cq values of the candidate reference genes obtained by qRT-PCR (Claus et al., 2004) and sorted the stability of the candidate reference genes according to the SD values and CV values (**Table 2**). To sum up, the lower the SD value, the more stable expression of this gene, the more suitable as an internal reference gene. As shown in **Table 2**, the SD value of *UBQ* was 0.95, the SD value of *RPS-18* was 1.23, and the SD value of *TBP* was 1.40. These genes were the top three stable genes expressed in different developmental stages of water dropwort and can be appropriately selected as internal reference genes. *GAPDH* has the highest SD value and was the most unstable gene at all stages. Based on BestKeeper software analysis, *UBQ*+*RPS-18* were selected as the internal reference gene in different developmental stages of water dropwort more appropriate.

#### 3.3.4 RefFinder analysis

There are some differences in the stability analysis results of the three algorithms for 10 candidate reference genes. Therefore, in order to obtain more accurate and reliable analysis results, we combined the results of various statistical algorithms by using RefFinder (Xie et al., 2012) to comprehensively evaluate the stability of 10 candidate internal reference genes in water dropwort (**Table 2**). The results showed that *TBP* and *UBQ* were the most stable reference genes in different developmental stages of water dropwort, while *GAPDH* was the least stable among all the reference genes. Through the comprehensive analysis of four software, it is more appropriate to choose *TBP*+*UBQ* as the internal reference genes in different developmental stages of water dropwort.

### 3.4 Determination of the optimum number of internal reference genes

**Figure 5:**
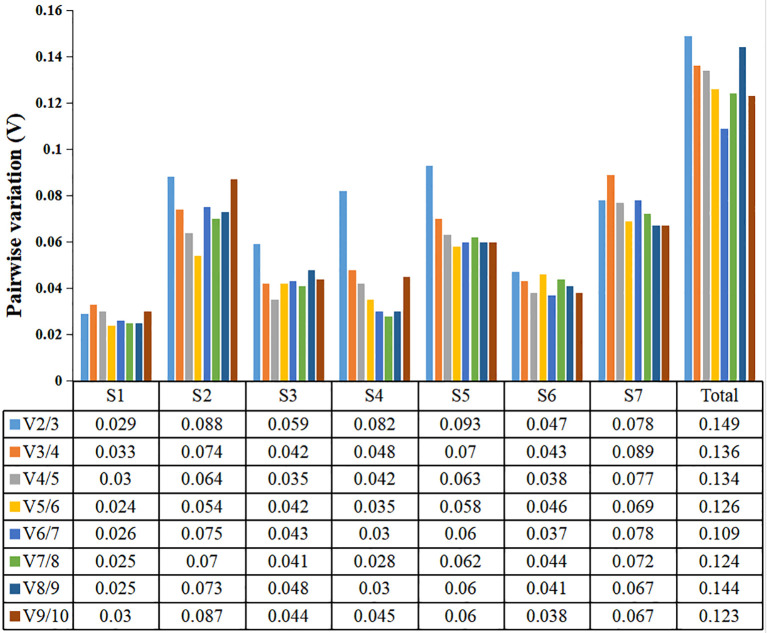
geNorm analysis of paired variation V values of ten candidate reference genes. Vn/n + 1 values are used to determine the optimal number of reference genes. S1-S7: The seven stages of water dropwort development. Total represents samples from the above seven experimental conditions.

The paired variation V values of candidate reference genes at different developmental stages were calculated by geNorm software, and the optimal number of required reference genes was determined according to the values of Vn/n +1 (Jo et al., 2002). When Vn/n +1> 0.15, the optimal number of internal reference genes is n+1; otherwise, only n internal reference genes were required. In this study, V2/3 of the seven developmental stages were all less than 0.15, and all subsequent data were less than 0.15 (**Figure 5**). Therefore, when analyzing the expression of key genes in seven different developmental stages of water dropwort in this study, two internal reference genes should be selected for normalization of target genes in order to be accurate.

### 3.5 Validation of reference genes

**Figure 6:**
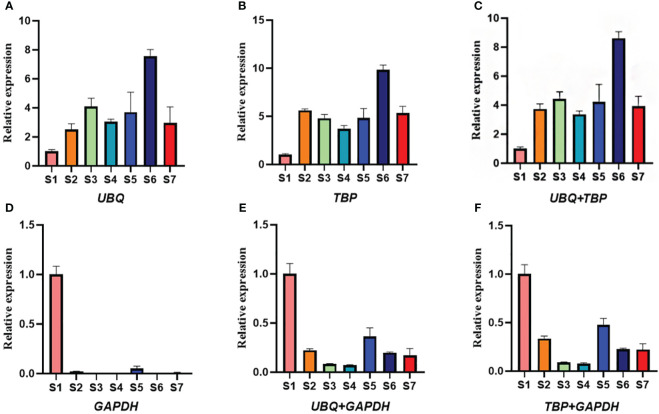
The relative expression levels of *EXP1* gene normalized by different internal reference genes. **(A)** The relative expression of *EXP1* when *UBQ* is used as an internal reference gene. **(B)** The relative expression of *EXP1* when *TBP* is used as an internal reference gene. **(C)** The relative expression of *EXP1* when *UBQ* and *TBP* are used as internal reference genes. **(D)** The relative expression of *EXP1* when *GAPDH* is used as an internal reference gene. **(E)** The relative expression of *EXP1* when *UBQ* and *GAPDH* are used as internal reference genes. **(F)** The relative expression of *EXP1* when *TBP* and *GAPDH* are used as internal reference genes. S1-S7: The seven stages of water dropwort development.

*EXP1* (encoding one expansion) is the major factor regulating cell wall extension and plays an important role in plant organ expansion (Wu et al., 2001). During the development of leaf and stem in water dropwort, the relative expression of *EXP1* showed an increasing trend with the continuous development and expansion of leaf and stem in water dropwort (Tang et al., 2023). To verify the stability of the two selected reference genes *TBP* and *UBQ*, the expression patterns of *EXP1* at different developmental stages were analyzed. When *UBQ* and *TBP* were used as internal reference genes alone for the normalized expression of *EXP1*, the results were consistent with expectations. The expression level of *EXP1* showed an increasing trend with the continuous development of water dropwort and reached a peak value in the S6 period (**Figure 6**). The relative expression level of *EXP1* also showed an upward trend when *UBQ* and *TBP* were joint acted as internal reference genes to normalize. However, the expression of *EXP1* showed a downward trend when the least stable internal reference gene *GAPDH* was used. In a word, two internal reference genes need to be selected for the normalization of target genes at different developmental stages of water dropwort, and the accuracy of screening results cannot be fully verified by selecting only the most unstable gene as the internal reference. Therefore, we chose to combine a stable gene and an unstable gene to normalize *EXP1* expression. No matter the combination of *TBP* and *GAPDH* or *UBQ* and *GAPDH*, the expression of target genes showed a downward trend with the continuous development of water dropwort. These results indicated that the reference genes we screened in different developmental stages of water dropwort are reliable.

## 4 Discussion

At present, gene expression analysis is a commonly used method to recognize the regulatory genes and molecular mechanisms in plants (Bustin et al., 2005; Silva et al., 2019). However, the accuracy of gene expression results is affected by the selected reference genes. Selecting the appropriate reference gene is essential for the normalization of target gene in qPCR (Udvardi et al., 2008). A variety of reference genes with stable and reliable expression have been reported in plants. These reference genes may express instability in different plants or genotypes (Wang et al., 2014; Wang et al., 2015). For instance, *GAPDH* is most stable gene in grapes, but ranks worst in wheat (Reid et al., 2006; Long et al., 2010). *TUB* is most stable in carrots, but less stable in raspberries and blackberries (Tian et al., 2015; Wu et al., 2021). Previous experiments have screened the most suitable endogenous reference gene for water dropwort under abiotic stress (Jiang et al., 2014), but the expression stability of endogenous reference gene has been confirmed to be different in different developmental stages and environmental stress (Libault et al., 2008).

Due to the large amount of sequence data obtained in water dropwort, the expression pattern and function analysis of many genes will be more convenient. The leaves and stems, as vegetative organs, are the products of specific developmental stages of water dropwort. Previous studies have revealed that several key genes are involved in regulating the synthesis of β-Caryophyllene, the main flavor substance in water dropwort (Feng et al., 2023b). The expression patterns and functional analysis of these genes in different tissues and tissue development stages of water dropwort need to be further studied. Therefore, in order to guarantee the accuracy and reliability of further study on the expression patterns and functional analysis results of key genes in different developmental stages of water dropwort. We need to screen the reference genes with the most stable expression levels in different growth and developmental stages of the water dropwort.

In this study, 10 candidate reference genes with stable performance in different species were selected based on previous experimental results (*ACTIN*, *PP2A*, *SAND*, *EF-1α*, *GAPDH*, *UBQ*, *MIP*, *TBP*, *RPS-18*, and *eIF-4α*) were selected (Libault et al., 2008; Wan et al., 2011; Galli et al., 2013; Kozera and Rapacz, 2013; Monteiro et al., 2013). RT-qPCR was used to detect the expression levels of candidate reference genes at different developmental stages. The melting curve verified the specificity and amplification efficiency of the primers. At the same time, the standard curves of 10 candidate reference genes were constructed in this study. According to the standard curves, the amplification efficiency (E) of 10 candidate reference genes was between 91.9 and 110%, and the correlation coefficient (R^2^) was greater than 0.99. The results showed that the amplification efficiency and primer specificity of each candidate reference gene met the conditions of subsequent qRT-PCR.

The expression was determined by qPCR as quantitative period (Cq) value. Cq values were used to evaluate gene expression levels. There were significant differences in the range of Cq values of 10 candidate reference genes in different developmental stages of water dropwort. Generally, Cq value between 15 and 35 is considered appropriate and valid data (Czechowski et al., 2005; Derveaux et al., 2010). Except the highest value of *SAND* gene was 36.37, the Cq values of all the other candidate reference genes ranged from 18 to 35. Among all candidate reference genes, *UBQ* gene had the highest average expression level, and the average Cq value was 21.49. The mean expression level of *SAND* gene was the lowest and the mean Cq value was 31.49. The Cq value of *UBQ* gene was 18.74-24.39 and the expression level of *UBQ* gene changed the least among all candidate reference genes. The expression level of *MIP* gene changed the most and its Cq value was 23.20-33.40. There were significant differences in the expression levels of candidate reference genes at different developmental stages. Their expression patterns were not completely stable in all samples. Therefore, we further used four statistical algorithms to evaluate the expression stability of 10 candidate reference genes at different developmental stages of water dropwort.

In our study, geNorm sorted the stability of candidate reference genes by calculating their M values (Jo et al., 2002). According to the ranking, *ACTIN*, *TBP* and *eIF-4α* as the internal reference genes with high expression stability in the seven developmental stages of water dropwort. However, the NormFinder algorithm based on the stability (S) value of the candidate internal reference genes and the BestKeeper algorithm based on the standard deviation (SD) and coefficient of variation (CV) of the candidate internal reference genes gave different results. Both NormFinder (Lund et al., 2011) and BestKeeper (Claus et al., 2004)listed *UBQ* as the most stable expression reference gene in the developmental stage of water dropwort, while *TBP* and *RPS-18* were reference genes with high expression stability. Although the three algorithms differed in the selection of genes with the most stable expression levels, they all listed *GAPDH* as the most unstable gene in the developmental of water dropwort. In order to obtain more accurate and reliable analysis results, we combined the results of various statistical algorithms, and used RefFinder (Xie et al., 2012) to comprehensively evaluate the stability of 10 candidate reference genes in water dropwort. The results showed that *TBP* and *UBQ* were the most stable genes, while *GAPDH* was the most unstable genes. The use of a single internal reference gene for calibration and standardization is thought to affect the accuracy of results (Zhu et al., 2008). It is generally believed that two or more internal reference genes can help to calibrate systematic bias (Schmid et al., 2003). We calculated the paired variant V values of candidate reference genes at different developmental stages of water dropwort by geNorm software, and determined the optimal number of required reference genes according to the values of Vn/n +1. In this study, the V2/3 of the seven developmental stages were all less than 0.15, and all subsequent data were less than 0.15. Therefore, when analyzing the expression of key genes in seven different developmental stages of water dropwort in this study, need selected two appropriate internal reference gene to achieve accurate normalization.

*EXP1* encoded a class of expansions, which contributed to cell expansion during the development of plant tissue (Zhou et al., 2020). In *Psidium guajava*, the expression of *EXP1* increased with fruit ripening and reached the highest level at the ripening stage (Silva et al., 2013). In *Solanum tuberosum*, *EXP* was closely related to the tuber expansion process, and the *EXP* gene exhibited high expression in growing tubers (Mao, 2013). In order to verify the reliability of internal reference genes, the most stable genes *TBP* and *UBQ* and the least stable genes *GAPDH* were selected to normalize the relative expression levels of *EXP1* genes. The results showed that when *UBQ* and *TBP* were normalized alone or in combination, the expression of *EXP1* increased with the continuous development of water dropwort, and reached a peak at S6 stage. However, when the least stable gene *GAPDH* was used for normalization, the expression of *EXP1* showed a downward trend. These results further confirm the reliability of the internal reference genes screened in this study and suggest that the use of inappropriate internal reference genes may lead to inaccurate results.

## 5 Conclusions

The purpose of this study was to screen out the reference genes of water dropwort with stable expression at different developmental stages. The stability of 10 candidate reference genes at different developmental stages was evaluated by geNorm, NormFinder, BestKeeper and RefFinder. The results showed that *UBQ* and *TBP* were the most stable internal reference gene, while *GAPDH* was the least stable gene in different developmental stages of water dropwort. The expression normalization of *EXP1* genes at different developmental stages verified the reliability of the reference genes in this study. In summary, the results of this study provide a theoretical basis for the selection of appropriate internal reference genes in different developmental stages of water dropwort. These results provided technical support and reliable basis for the expression analysis of key genes in different developmental stages of water dropwort.

## Data availability statement

The original contributions presented in the study are included in the article/**Supplementary Material**, further inquiries can be directed to the corresponding author.

## Author contributions

KF: Conceptualization, Funding acquisition, Supervision, Writing – review & editing. Z-YY: Data curation, Formal analysis, Methodology, Writing – original draft. Y-JY: Data curation, Writing – review & editing. NS: Investigation, Writing – original draft. Z-QZ: Data curation, Writing – review & editing. J-LL: Data curation, Writing – review & editing. S-PZ: Resources, Writing – review & editing. PW: Investigation, Writing – review & editing. L-JL: Data curation, Funding acquisition, Resources, Supervision, Writing – review & editing.
