# Supplementary material for: Selection of suitable reference genes for qPCR normalization in different developmental stages of Oenanthe javanica
Source: Front Plant Sci. 2023 Dec 27;14:1287589. doi: 10.3389/fpls.2023.1287589 (PMC10777208; doi:10.3389/fpls.2023.1287589)
Supplement: Supplementary file 1 [file DataSheet_1.docx]

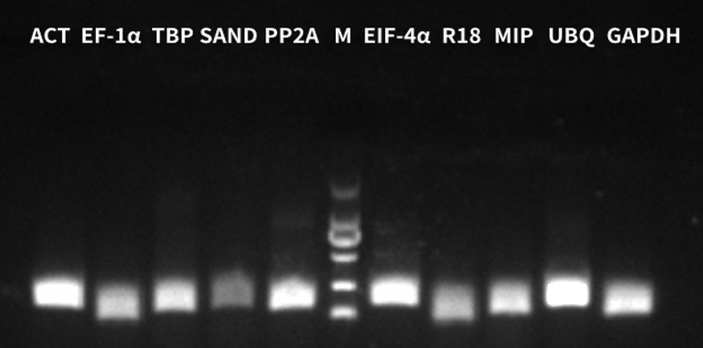


**Figure S1** Ten candidate reference gene primers were amplified by qPCR.


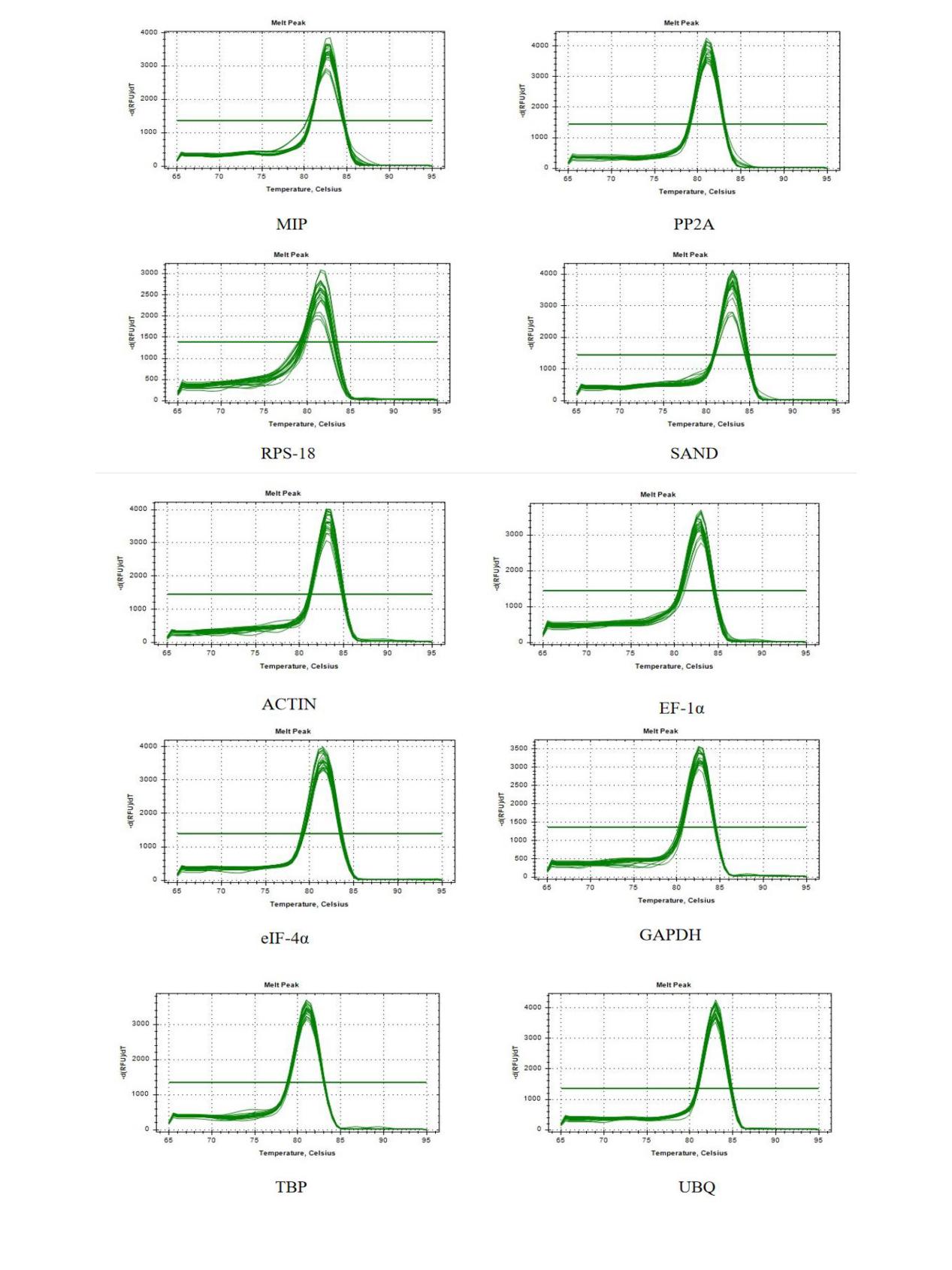


**Figure S2** Melting curves generated for ten candidate reference genes by qPCR.


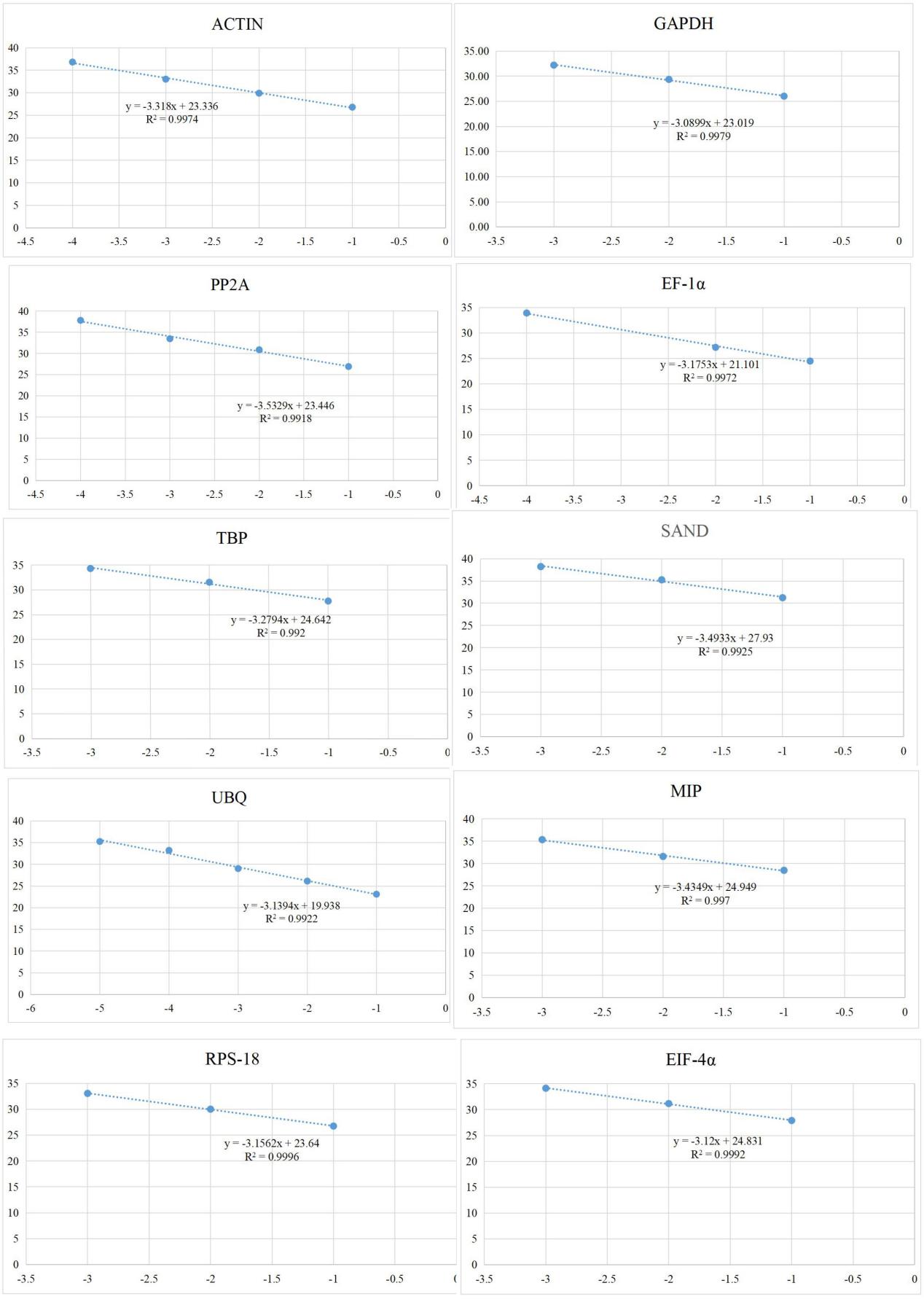


**Figure S3** Standard curves of ten candidate reference genes in different developmental stages of water dropwort.

1 atgattaagcagatatttggtaaacggaagacatcgtctaaatcatcctcgcaatatgattcgagtaatgatgttgggtattcatcgata

M I K Q I F G K R K T S S K S S S Q Y D S S N D V G Y S S I

91 aatacattgaattcagctggtggggttgctaataatttgactaagactagttctgcatctgggaaaggttcgaattctgcttctgcaact

N T L N S A G G V A N N L T K T S S A S G K G S N S A S A T

181 ttgaaatcgagtaatgggaactatggtccccaagtgtcgagtgctgctgcacttgtatcgaatcaagggaagaagtcgggtcaagtgggt

L K S S N G N Y G P Q V S S A A A L V S N Q G K K S G Q V G

271 tcccaagttggtcaggggatgaattatggtgtttatgaagctttgcctatctttcgggatgttcccagctctgaaaagcagaatctgttt

S Q V G Q G M N Y G V Y E A L P I F R D V P S S E K Q N L F

361 attaggaagttggatatgtgttgtggtgttgtttgattttagtgatgcttcgaagaatgttaaagagaaggagataaaacgacagacttt

I R K L D M C C G V V * F * * C F E E C * R E G D K T T D F

451 gcttgaacttgttgactacatttcgtcagcaaattcgaagtttaatgagattgccatgcaggagatcacgaaaatggtagctgcgaatct

A * T C * L H F V S K F E V * * D C H A G D H E N G S C E S

541 ttttcgagcatttgcatctagtcatgataacaaactaccagatgtgtatgacccagaggatgatgaaccagctatggaaccatcatggcc

F S S I C I * S * * Q T T R C V * P R G * * T S Y G T I M A

631 tcatcttcaaattgtgtatgagtttcttttgagatttgtggcttctactgaaacagatgcaaaacttgcaaaaagatatattgaccattc

S S S N C V * V S F E I C G F Y * N R C K T C K K I Y * P F

721 ctttgtgttgagattacttgatctttttgactcggacgatcaacgagagagggagtacttgaagacaattctacatcgtatatatgggaa

L C V E I T * S F * L G R S T R E G V L E D N S T S Y I W E

811 gttcatggtgcaccggccatttattaggaaatccatcaacaatattttttacaattttatatctgagactgtgcgacacaatgggattgc

V H G A P A I Y * E I H Q Q Y F L Q F Y I * D C A T Q W D C

901 tgaattgcttgaaatcttgggtagtataattaatgggtttgctctccctttaaaagaagaacacaagcttttccttgttcgtgcactgat

* I A * N L G * Y N * W V C S P F K R R T Q A F P C S C T D

991 tccccttcacaaacctaaaagtgtatcaatgtaccaccagcaactttcgtattgtattacccaatttgtggagaaggatattaagctggc

S P S Q T * K C I N V P P A T F V L Y Y P I C G E G Y * A G

1081 tgacacagtaattcgagggttgttgaagtactggccgataactaatagttcaaaagaagtcatgttccttggtgaattggaggaagttct

* H S N S R V V E V L A D N * * F K R S H V P W * I G G S S

1171 ggaagctacccaggctgcagaatttcaaaagtgcatggtccgtctgttccgtcagataggtcgctgcctcaacagctcacatttccaggt

G S Y P G C R I S K V H G P S V P S D R S L P Q Q L T F P G

1261 agctgagcgcacattgtttttatggaataacgatcatataaggactctgattacacagaatcgcaagatcatactgccaattattttccc

S * A H I V F M E * R S Y K D S D Y T E S Q D H T A N Y F P

1351 agccttggagaataatactcggagtcactggaaccaagctgtccatagtttgacgctgaatgtgagaaaaatattcatagacgcagatca

S L G E * Y S E S L E P S C P * F D A E C E K N I H R R R S

1441 agcactttttgatgagtgcttagctaagttccaagaagatgagatgaaagagaaaaatatacttgagaagcgtgaatcaacttggaagcg

S T F * * V L S * V P R R * D E R E K Y T * E A * I N L E A

1531 cttggaagatatggcagcatccaaggctgtaagcaatgaggcagtgcttgtctcaaaattttcttccgttgacattgtcgcaagcacaaa

L G R Y G S I Q G C K Q * G S A C L K I F F R * H C R K H K

1621 ttcaccgaagaccgccggtagttga

F T E D R R * L X

**Fig. S4** Nucleotide acid and deduced amino acid sequence of *PP2A* gene from *Oenanthe javanica*.

1 atggccgacggtgaagatattcaacctcttgtttgcgataatggaactggaatggtcaaggctggatttgctggcgatgatgctccaagg

M A D G E D I Q P L V C D N G T G M V K A G F A G D D A P R

91 gctgtgttccccagtattgttggcagacctcggcacactggtgttatggttggaatgggccaaaaggatgcctatgttggagatgaagca

A V F P S I V G R P R H T G V M V G M G Q K D A Y V G D E A

181 caatcaaaaaggggtatcctgacattgaaatacccaattgaacatggcattgttagcaattgggatgacatggagaagatatggcatcac

Q S K R G I L T L K Y P I E H G I V S N W D D M E K I W H H

271 actttctataatgagctgcgtgtagcccctgaagagcacccagtattacttacagaagcaccactgaatcctaaggcaaacagggaaaaa

T F Y N E L R V A P E E H P V L L T E A P L N P K A N R E K

361 atgacccaaattatgtttgagaccttcaacgtgcctgccatgtatgttgctatccaggccgtgctgtctctatatgctagtggccgtaca

M T Q I M F E T F N V P A M Y V A I Q A V L S L Y A S G R T

451 acaggtattgttttggactctggtgatggtgtaagccacacggtgccaatatatgaaggctatgcacttccacatgcaattcttcgacta

T G I V L D S G D G V S H T V P I Y E G Y A L P H A I L R L

541 gaccttgctggtcgtgatcttacagatggcttgatgaagattctcacagaaagaggttatatgttcactaccactgctgaacgggaaatc

D L A G R D L T D G L M K I L T E R G Y M F T T T A E R E I

631 gttcgtgatatgaaggagaacttgcctatgtcgctcttgattatgagcaagagttggagactgccaagagcagctctgctgtcgagaaaa

V R D M K E N L P M S L L I M S K S W R L P R A A L L S R K

721 actatgaacttcctgatgggcaggttatcaccattggagctgagaggttccgctggtccagaagtcctgttccagccatctatgattgga

T M N F L M G R L S P L E L R G S A G P E V L F Q P S M I G

811 atggaagctgccggaatccatgaaactacttacaactctattatgaagtgtgatgttgatatcagaaaagatctttatggtaatattgtc

M E A A G I H E T T Y N S I M K C D V D I R K D L Y G N I V

901 cttagtggtgggtcaactatgttccctggtattgctgatcgtatgagcaaagaaatcactgcccttgctcccagtagcatgaagatcaag

L S G G S T M F P G I A D R M S K E I T A L A P S S M K I K

991 gtggtggcgcctccagaaagaaagtacagtgtttggattggaggatcgatcctcgcttctctcagcaccttccagcagatgtggatctcc

V V A P P E R K Y S V W I G G S I L A S L S T F Q Q M W I S

1081 aagggtgaatatgacgaatcaggtccatcaattgttcatcgaaagtgcttctaa

K G E Y D E S G P S I V H R K C F *

**Fig. S5** Nucleotide acid and deduced amino acid sequence of *ACTIN* gene from *Oenanthe javanica*.

1 atggcctctaatgcagctcttgcttcgtcaagaattccgaccactaccagtcttccttccaaaagctctcattcttaccctactcaatgc

M A S N A A L A S S R I P T T T S L P S K S S H S Y P T Q C

91 ttctccaagagactcgatgtagctgaattttctgggcttcgatccagtgtttccctgacatactctaggagtgctagagaaggatctttc

F S K R L D V A E F S G L R S S V S L T Y S R S A R E G S F

181 tttgatgtggtggctgcacaactgactcctaagactacaggaacattgcctgttaaaggagagacagttgccaaattgaaagttgcaatc

F D V V A A Q L T P K T T G T L P V K G E T V A K L K V A I

271 aatggttttggtcgcataggcagaaacttcctccgctgctggcatgggcgcaaagactctccacttgaagtcgtagttctcaatgacagt

N G F G R I G R N F L R C W H G R K D S P L E V V V L N D S

361 ggtggtgtcaagaatgcatcgcacttgctgaagtatgactctatgttgggtactttcaaggccgatgtaaagatagtagacaatgaaacc

G G V K N A S H L L K Y D S M L G T F K A D V K I V D N E T

451 atcagtgttgatggaaagctgatcaaggttgtctccaacagagaccctctccagcttccatgggcagagcttggtattgacattgttatc

I S V D G K L I K V V S N R D P L Q L P W A E L G I D I V I

541 gaggggacaggtgtgtttgtggatggccctggcgctgggaaacacatccaagccggtgccaagaaagttattatcactgcaccagcgaaa

E G T G V F V D G P G A G K H I Q A G A K K V I I T A P A K

631 ggagctgatattccaacatatgttgttggagtaaatgaaaaggaatatgatcatgatgttgcaaatattgtcagcaatgcttcttgcaca

G A D I P T Y V V G V N E K E Y D H D V A N I V S N A S C T

721 accaactgtttggctccatttgcaaaggtccttgatgatgaattcggtatcgtcaaaggaacaatgacgaccactcattcctacactggt

T N C L A P F A K V L D D E F G I V K G T M T T T H S Y T G

811 gaccagaggcttttggatgcttcacacagagacttgaggagagccagagctgcagcactgaatatagtcccaacaagtactggtgcagcc

D Q R L L D A S H R D L R R A R A A A L N I V P T S T G A A

901 aaggcagtgtctttagttctaccccagcttaagggcaagctcaatggtattgctctacgggtgcccactccaaatgtatccgttgttgac

K A V S L V L P Q L K G K L N G I A L R V P T P N V S V V D

991 cttgttgtgaatgttgcaaagaaaggaatttcagccgaagatgtcaatgctgcattcagaaaggcagctgatggaccaatgaaggggatt

L V V N V A K K G I S A E D V N A A F R K A A D G P M K G I

1081 ctagctgtatgcgatgagcctctcgtttcagtagactttcggtgctctgatgtttccaccaccattgacgcatcgttgtcaatggtcatg

L A V C D E P L V S V D F R C S D V S T T I D A S L S M V M

1171 ggagatgatatggtcaaggttgtagcctggtacgacaacgaatggggttacagccaaagagttgttgatctggcacatcttgtagcaagc

G D D M V K V V A W Y D N E W G Y S Q R V V D L A H L V A S

1261 aaatggccaggtgcagcagcaggaggaagtggagaccctctggaggattactgcaaatcaaaccctgctgaagaggagtgcaaagttttt

K W P G A A A G G S G D P L E D Y C K S N P A E E E C K V F

1351 gattaa

D *

**Fig. S6** Nucleotide acid and deduced amino acid sequence of *GAPDH* gene from *Oenanthe javanica*.

1 atgggtaaggaaaaggttcacatcaacattgtggtcattggccatgtggactctggaaaatcgaccaccactggtcacttgatctacaag

M G K E K V H I N I V V I G H V D S G K S T T T G H L I Y K

91 cttggtggtattgacaagcgtgttattgagagatttgagaaagaagctgctgaaatgaacaagaggtctttcaagtatgcttgggtgctt

L G G I D K R V I E R F E K E A A E M N K R S F K Y A W V L

181 gacaagttgaaggctgagcgagaacgtggtattaccattgatatcgccttgtggaagtttgaaaccaacagatactactgcactgtcatt

D K L K A E R E R G I T I D I A L W K F E T N R Y Y C T V I

271 gatgcccctggacatcgtgactttatcaagaatatgattactggaacatctcaggctgattgtgctgttctaatcattgactctactact

D A P G H R D F I K N M I T G T S Q A D C A V L I I D S T T

361 ggtggttttgaagctggtatttccaaggatggtcagacccgtgaacatgctttgctcgctttcaccctcggtgtcaaacaaatgatttgt

G G F E A G I S K D G Q T R E H A L L A F T L G V K Q M I C

451 tgctgtaacaagatggatgccaccaccccaaaatactccaaggccaggtatgatgaaattgtgaaggaggtttcttcgtacttgaagaag

C C N K M D A T T P K Y S K A R Y D E I V K E V S S Y L K K

541 gttgggtacaaccctgataaaattgcttttgtccccatttctggatttgagggtgataacatgattgagaggtccacaaacttggactgg

V G Y N P D K I A F V P I S G F E G D N M I E R S T N L D W

631 tacaagggccctactcttcttgaggcccttgaccagatcaatgagcccaaaagaccctcagacaagccccttcgtctcccacttcaggat

Y K G P T L L E A L D Q I N E P K R P S D K P L R L P L Q D

721 gtgtacaagattggtggtattggaactgtgccagtggggagagttgagactggtactctgaagcctggaatggttgtcacttttggcccc

V Y K I G G I G T V P V G R V E T G T L K P G M V V T F G P

811 tctgggctgaccactgaagttaagtctgttgagatgcatcatgagtctcttctagaggcacttcctggtgacaatgttggcttcaatgtc

S G L T T E V K S V E M H H E S L L E A L P G D N V G F N V

901 aagaatgttgctgtcaaggatctcaagcgtgggtatgtggcttcaaactccaaggatgatccagccaagggagctgccaatttcacatct

K N V A V K D L K R G Y V A S N S K D D P A K G A A N F T S

991 caggtcatcatcatgaaccaccctggacagattggtaacggatatgctccagtcctcgattgtcatacctcccacattgctgtcaaattt

Q V I I M N H P G Q I G N G Y A P V L D C H T S H I A V K F

1081 gctgagctcttgaccaagattgacaggcgatctggaaaggagctcgagaaggagcccaagttcttgaagaatggtgatgctgggatggtc

A E L L T K I D R R S G K E L E K E P K F L K N G D A G M V

1171 aagatgcttccaaccaagcccatggtggtggagactttttccgagtaccctcctcttggaaggtttgctgttcgtgatatgagacagaca

K M L P T K P M V V E T F S E Y P P L G R F A V R D M R Q T

1261 gttgccgttggtgtcatcaaggctgttgacaagaaggatccaactggtgccaaggttaccaaggcagcagcaaagaagggtgccaagtga

V A V G V I K A V D K K D P T G A K V T K A A A K K G A K *

1351

**Fig. S7** Nucleotide acid and deduced amino acid sequence of *EF-1α* gene from *Oenanthe javanica*.

1 atggctggagctgcaccagaaggttctcaattcgatgcacgtcaatttgacgcaaaaatgactgagctacttggtgctgatggagaagaa

M A G A A P E G S Q F D A R Q F D A K M T E L L G A D G E E

91 ttctttacatcatatgatgaggtttatgacagttttgatgctatgggattgcaggaaaatcttttgagaggcatctatgcttacggtttt

F F T S Y D E V Y D S F D A M G L Q E N L L R G I Y A Y G F

181 gagaagccatctgcgattcagcagcggggtattgttccattctgcaaagggctagatgttattcaacaggcacaatctggtactgggaaa

E K P S A I Q Q R G I V P F C K G L D V I Q Q A Q S G T G K

271 acagcaactttctgctctggagttctgcagcagcttgattttgctaatgttgaatgtcaagcattggttcttgctcctactcgtgaattg

T A T F C S G V L Q Q L D F A N V E C Q A L V L A P T R E L

361 gcacaacagattgagaaggttatgcgagcacttggtgattatcttggtgtgaaggttcatgcctgtgttggtggaacccagtgtccgtga

A Q Q I E K V M R A L G D Y L G V K V H A C V G G T Q C P *

451 agatcagcgcattctctccagtggagttcatgttgtggttggtactcctggtcgtgtattcgacatgttgcgaagacagtctctgcgctc

R S A H S L Q W S S C C G W Y S W S C I R H V A K T V S A L

541 agattacatcaagatgtttgttctggacgaagcagatgaaatgctttcaagaggatttaaggatcagatttatgatatcttccagttgtt

R L H Q D V C S G R S R * N A F K R I * G S D L * Y L P V V

631 acctcctaaagttcaggttggtgttttctctgccaccatgcctcccgaggctcttgaaattactaggaaattcatgaataagcctgtgag

T S * S S G W C F L C H H A S R G S * N Y * E I H E * A C E

721 gattcttgtaaagcgagatgagctcactctcgagggtatcaaacaattttatgttaatgttgacaaggaggaatggaaattggaaacact

D S C K A R * A H S R G Y Q T I L C * C * Q G G M E I G N T

811 ttgtgatctttatgagactttagctattacacagagtgtcatctttgttaataccaggcgcaaggttgattggctgactgacaaaatgcg

L * S L * D F S Y Y T E C H L C * Y Q A Q G * L A D * Q N A

901 cagtcgtgatcacacagtctctgccactcatggagacatggatcagaatactagagatataattatgcgtgagtttcgctctggttcttc

Q S * S H S L C H S W R H G S E Y * R Y N Y A * V S L W F F

991 tcgtgtgctcattaccactgatctcctggctcgtggtatagatgtccagcaagtatcgcttgtgataaactatgatctgccgactcagcc

S C A H Y H * S P G S W Y R C P A S I A C D K L * S A D S A

1081 agagaactacctccatcgaattggacgtagtggacgttttggaaggaaaggtgttgcaatcaactttgtgaccaaggatgacgacaggat

R E L P P S N W T * W T F W K E R C C N Q L C D Q G * R Q D

1171 gttgtttgacatacagaagttttataatgtagtagtggaggagcttccagctaatgttgccgatcttctttag

V V * H T E V L * C S S G G A S S * C C R S S L X

**Fig. S8** Nucleotide acid and deduced amino acid sequence of e*IF-4α* gene from *Oenanthe javanica*.

1 atggcggaacagggaatggaaggtagccaaccagtggatctttccaagcatccatctggaattgtacccactcttcagaatatagtatca

M A E Q G M E G S Q P V D L S K H P S G I V P T L Q N I V S

91 actgtcaacttggattgcaagttagatttgaaagccattgcattgcaagcgagaaatgctgaatataaccccaagcgttttgctgctgtc

T V N L D C K L D L K A I A L Q A R N A E Y N P K R F A A V

181 attatgcgaatcagggatccaaagacaacagcacttatatttgcttcgggaaaaatggtttgtactggagcaaagagtgaacaacagtca

I M R I R D P K T T A L I F A S G K M V C T G A K S E Q Q S

271 aaattggctgcaaggaagtatgcacgaattattcaaaagcttagttttcctgccaaattcaaggactttaagatacaaaatattgttggt

K L A A R K Y A R I I Q K L S F P A K F K D F K I Q N I V G

361 tcttgtgatgtgaaatttcctatcagacttgaaggtcttgcatattcccatggtgccttttctagttatgaaccagaactatttcctggt

S C D V K F P I R L E G L A Y S H G A F S S Y E P E L F P G

451 ctaatatatcgtatgaagcaacccaagattgtcctcctcatcttcgtgtcgggaaaaattgtacttaccggagcaaaggtgagagaagaa

L I Y R M K Q P K I V L L I F V S G K I V L T G A K V R E E

541 acgtatacagcatttgagaacatatatcctgttcttacagagttcagaaagaatcagcagtga

T Y T A F E N I Y P V L T E F R K N Q Q *

**Fig. S9** Nucleotide acid and deduced amino acid sequence of *TBP* gene from *Oenanthe javanica*.

1 atgcagatatttgtgaaaaccttgacgggaaagacaatcaccttggaggtggaaagctccgacacaatcgataacgtcaaggccaaaatc

M Q I F V K T L T G K T I T L E V E S S D T I D N V K A K I

91 caggacaaggaaggcattcctcctgaccagcaacgtctcatcttcgctggtaaacagttggaagatggccgaactctggctgattacaac

Q D K E G I P P D Q Q R L I F A G K Q L E D G R T L A D Y N

181 atccagaaggagtccacacttcacctcgtgctccgtctccgtgggggtatgcagatttttgtcaagaccttgacagggaaaactattact

I Q K E S T L H L V L R L R G G M Q I F V K T L T G K T I T

271 ctcgaagttgagagctctgatacgatcgacaatgtcaaggccaagattcaggacaaggagggaattcctccagaccagcagcgtttgatt

L E V E S S D T I D N V K A K I Q D K E G I P P D Q Q R L I

361 ttcgccggaaagcagttggaggatggcaggaccctcgccgactataacatccagaaggagtctacactccatcttgtgttgcgtctccgt

F A G K Q L E D G R T L A D Y N I Q K E S T L H L V L R L R

451 ggtggtatgcagatctttgtcaagaccctgactggcaagacaatcactcttgaagttgagagctctgacactattgacaatgtcaaggcc

G G M Q I F V K T L T G K T I T L E V E S S D T I D N V K A

541 aagatccaggacaaggaagggattcccccagaccagcagcgtctcatctttgctgggaagcaattggaagatggcaggacccttgcagat

K I Q D K E G I P P D Q Q R L I F A G K Q L E D G R T L A D

631 tataacattcagaaggagtccactctccaccttgtgttgcgtcttcgtggtggtatgcagattttcgtcaagactctgaccggaaagacc

Y N I Q K E S T L H L V L R L R G G M Q I F V K T L T G K T

721 atcaccctggaggtggagagttcggataccattgacaatgtcaaggccaagatccaggataaggaagggattcctccagatcagcagagg

I T L E V E S S D T I D N V K A K I Q D K E G I P P D Q Q R

811 ttgatctttgctgggaagcagctagaagatgggaggacattggcagactataacatccagaaggagtccactcttcacttggtgttgcgt

L I F A G K Q L E D G R T L A D Y N I Q K E S T L H L V L R

901 ctccgtggtggtgatttctga

L R G G D F *

**Fig. S10** Nucleotide acid and deduced amino acid sequence of *UBQ* gene from *Oenanthe javanica*.

1 atgttatcagatgatgctaactcctcatcagatgacgactccactgaccaaaaccctaaccctagcacttcaatcgaccaatctctcgac

M L S D D A N S S S D D D S T D Q N P N P S T S I D Q S L D

91 gcaattgaaggtcaattgacctcaatttcactcaaccaccacgtttctccactcccagcgccctctattccgcctcctgatccccaatat

A I E G Q L T S I S L N H H V S P L P A P S I P P P D P Q Y

181 atagatacattgccttccgattcgtcttcacaagttgaacaagtactagtagttgaaaattccgtgtcagaaggaggtttagatacattt

I D T L P S D S S S Q V E Q V L V V E N S V S E G G L D T F

271 ccttctgaatcgtgtactctagtcgaagaagtagtagttgagaattcggtgttgtggaggaataattcggatgtggaagtcgaaggggat

P S E S C T L V E E V V V E N S V L W R N N S D V E V E G D

361 ggtcaggggagtccgagtagtagtggatatgctggcggtaaagggactagtagtagtgctttgagtggttcgggaattgaggagattagt

G Q G S P S S S G Y A G G K G T S S S A L S G S G I E E I S

451 ggcgatgagagtgatcacggtgagctagtcaaaaggagtggttcttttggtggtagtgtggattcggagtgggttcccgggaaacgacat

G D E S D H G E L V K R S G S F G G S V D S E W V P G K R H

541 gttaatgaagatgatgcttctgtttcatggaggaaaaggaagaaacatttttttatcttaagccattctggtaaaccaatatattcaaga

V N E D D A S V S W R K R K K H F F I L S H S G K P I Y S R

631 tatggagatgaacacagactagcaggattttcagcaactttgcaagctatcatttccttcgtggagatggggggagatcatgtgaagttg

Y G D E H R L A G F S A T L Q A I I S F V E M G G D H V K L

721 gttagggcgggaaacaccaaggtggtttttctcgttaaaggaccaatatatctagtttgcataagctgtacagaagagcctcatgaatcc

V R A G N T K V V F L V K G P I Y L V C I S C T E E P H E S

811 ctcaacgaacaactggaacttctttatgggcagatgatacttattttgacaaagtctataaatagatgctttgagaagatccaaatttga

L N E Q L E L L Y G Q M I L I L T K S I N R C F E K I Q I *

901 tatgacgcctttgcttgggggaacagatgctgtattctcctctctcatccactcggttagttgggagcctgccacttttcttccatggcc

Y D A F A W G N R C C I L L S H P L G * L G A C H F S S M A

991 gactctagtcttcccccttcttatgcaacaaggaggcgctggtgctatattgcaggatgttgctgagtcaggtgtcctctttgcaatatt

D S S L P P S Y A T R R R W C Y I A G C C * V R C P L C N I

1081 aatgtgtaaacacaaggtcatcagtctggttggtgcacaaaaagcatctcttcatcccgatgatatgctcttgcttgccaactttgtgat

N V * T Q G H Q S G W C T K S I S S S R * Y A L A C Q L C D

1171 gtcatctgaatcatttaggacttctgaatctttctctccaatctgtcttccgagatacaatccaatggcatttttatatgcttatgtata

V I * I I * D F * I F L S N L S S E I Q S N G I F I C L C I

1261 ttatcttgatgctgatacatatttgatgttgcttactgctaatccagatgcattttatcgtctaaaagattggaggatccgtattgaaat

L S * C * Y I F D V A Y C * S R C I L S S K R L E D P Y * N

1351 ggtccttctgaagtcaaatgttcttaatgaagctcagaggtcgatgttggatggtggcatgcatgttgaggatttgcctcttgatccatc

G P S E V K C S * * S S E V D V G W W H A C * G F A S * S I

1441 ttctcgccctggatctttgtcatctcatttaggtcagcctagacctccaccaggctctgcagatgggtgtaaggcactgttaggtggtcc

F S P W I F V I S F R S A * T S T R L C R W V * G T V R W S

1531 tgctgggctttggcacttcatttaccgcagtatatatctagatcaatatgtatcttctgaattctcatcaccaattaacactcctaaaca

C W A L A L H L P Q Y I S R S I C I F * I L I T N * H S * T

1621 acagaaaagattatatagagcatatcagaagctgtatacttctatgcatgatagagaacttggtcctcacaaaacccagtttagaaggga

T E K I I * S I S E A V Y F Y A * * R T W S S Q N P V * K G

1711 caataactatgttttactctgctgggttactcaggattttgaactttatgcagcatttgatcctctagcagacaaggcactggctataaa

Q * L C F T L L G Y S G F * T L C S I * S S S R Q G T G Y K

1801 gacatgcaaccgagtatgccaatgggttaaagatgtggagaatgaagtttttttgttgggagcaagccccttttcatggtga

D M Q P S M P M G * R C G E * S F F V G S K P L F M V X

**Fig. S11** Nucleotide acid and deduced amino acid sequence of *SAND* gene from *Oenanthe javanica*.

1 atggagggtaaagaagaggatgttaagcttggagctaacaagttctcagagaggcagcctctgggtacatctgcacagacagacaaggac

M E G K E E D V K L G A N K F S E R Q P L G T S A Q T D K D

91 tacaaggagccaccaccagcaccattgtttgagcctggcgaactcacatcatggtccttttacagggctgggattgctgagttcatagcc

Y K E P P P A P L F E P G E L T S W S F Y R A G I A E F I A

181 actttcttgttcttgtatgtcactgttttgactgttatgggtgtgtctagggcacctaataagtgtgcttctgtgggtattcaaggaatt

T F L F L Y V T V L T V M G V S R A P N K C A S V G I Q G I

271 gcttgggcttttggtggcatgattttcgcccttgtttactgcactgctggtatttcaggagggcacataaacccagctgtgacctttggt

A W A F G G M I F A L V Y C T A G I S G G H I N P A V T F G

361 ttgtttctggctaggaagctctcacttaccagggctctgttctatatggtgatgcagtgccttggtgccatttgtggtgctggtgttatc

L F L A R K L S L T R A L F Y M V M Q C L G A I C G A G V I

451 aagggcttcgagggatcttcaagatttgaactcaacggcggtggtgctaatgttgtgaaccatggctacacaaagggtgatggacttggt

K G F E G S S R F E L N G G G A N V V N H G Y T K G D G L G

541 gctgagattgttggcacttttgttcttgtgtacactgtcttctctgcgactgatgctaagagaagcgccagagactcgcacgttcctata

A E I V G T F V L V Y T V F S A T D A K R S A R D S H V P I

631 ttggctccactgcctattggatttgcagtgttcttggttcatttggccaccatccccatcacaggaactggtattaacccagctagaagt

L A P L P I G F A V F L V H L A T I P I T G T G I N P A R S

721 ctcggtgctgccatcatttacaacaaagatcacgcatgggatgatcactgggtcttctgggttggtcctttcattggtgctgctcttgct

L G A A I I Y N K D H A W D D H W V F W V G P F I G A A L A

811 gccttgtaccaccagatagtcatcagagccattcctttcaagagcagggcttga

A L Y H Q I V I R A I P F K S R A *

**Fig. S12** Nucleotide acid and deduced amino acid sequence of *MIP* gene from *Oenanthe javanica*.

1 atgtcgcttgtagcaaatgaagattttcagcacattcttcgtattcaaaacacgaatgttgatgggaagcagaagatcatgttcgctttg

M S L V A N E D F Q H I L R I Q N T N V D G K Q K I M F A L

91 acttcgattaaaggtatcggacgtcgtttcgcaaacattgtttgcaaaaaagctgacgtggatatgaataagagggctggtgaactttca

T S I K G I G R R F A N I V C K K A D V D M N K R A G E L S

181 tctgctgaaattgacagtttgatgaccatcgttgctaatcctcgacagttcaagatcccagattggttcttgaacagaaagaaggattac

S A E I D S L M T I V A N P R Q F K I P D W F L N R K K D Y

271 aaggatggaaagttctctcaggtgacatccaatgcactagacatgaaactaagagatgatcttgagcgcctgaagaaaattaggaaccat

K D G K F S Q V T S N A L D M K L R D D L E R L K K I R N H

361 cgtggtctccgtcactactggggtcttcgagtacgtggacaacacaccaagactacgggacgaaggggaaagactgttggtgtctctaag

R G L R H Y W G L R V R G Q H T K T T G R R G K T V G V S K

451 aagagatag

K R *

**Fig. S13** Nucleotide acid and deduced amino acid sequence of *RPS-18* gene from *Oenanthe javanica*.

Table S1 Cq values of candidate reference genes in RT-qPCR assay.

|  | Replicates | *ACTIN* | *PP2A* | *SAND* | *EF-1α* | *GAPDH* | *UBQ* | *MIP* | *TBP* | *RPS-18* | *eIF-4α* |
| --- | --- | --- | --- | --- | --- | --- | --- | --- | --- | --- | --- |
| S1-1 | 1 | 23.75 | 24.68 | 27.06 | 20.53 | 29.55 | 19.16 | 23.61 | 25.38 | 23.08 | 21.38 |
|  | 2 | 23.74 | 24.77 | 27.05 | 20.64 | 29.13 | 19.23 | 24.14 | 25.44 | 23.04 | 21.62 |
|  | 3 | 23.79 | 24.72 | 27.02 | 20.56 | 29.32 | 19.04 | 23.95 | 25.49 | 23.10 | 21.26 |
| S1-2 | 4 | 23.73 | 24.61 | 26.68 | 20.54 | 29.04 | 19.01 | 23.48 | 25.11 | 23.06 | 21.35 |
|  | 5 | 23.64 | 24.56 | 26.57 | 20.50 | 28.86 | 18.74 | 23.20 | 24.67 | 22.70 | 21.38 |
|  | 6 | 23.59 | 24.54 | 26.73 | 20.60 | 28.93 | 18.88 | 23.36 | 25.22 | 23.14 | 21.04 |
| S1-3 | 7 | 23.76 | 24.67 | 26.97 | 20.47 | 29.32 | 19.23 | 24.09 | 25.52 | 22.90 | 21.52 |
|  | 8 | 23.67 | 24.56 | 27.02 | 20.45 | 28.73 | 18.76 | 24.09 | 25.45 | 23.02 | 21.62 |
|  | 9 | 23.74 | 24.51 | 27.00 | 20.58 | 29.43 | 19.12 | 23.43 | 25.28 | 23.14 | 21.53 |
| S2-1 | 1 | 28.40 | 27.99 | 32.31 | 28.44 | 20.66 | 21.34 | 28.40 | 29.18 | 26.72 | 26.18 |
|  | 2 | 28.31 | 28.01 | 32.23 | 28.35 | 20.33 | 21.36 | 28.31 | 29.20 | 26.52 | 26.12 |
|  | 3 | 28.25 | 27.91 | 32.08 | 28.33 | 20.46 | 21.20 | 28.10 | 28.60 | 26.52 | 26.21 |
| S2-2 | 4 | 28.24 | 27.93 | 31.29 | 28.66 | 20.72 | 21.65 | 28.56 | 29.07 | 26.96 | 25.42 |
|  | 5 | 28.19 | 27.88 | 31.30 | 28.58 | 20.37 | 21.94 | 28.77 | 28.81 | 26.64 | 25.43 |
|  | 6 | 28.06 | 27.95 | 31.27 | 28.63 | 20.49 | 21.34 | 28.40 | 29.00 | 26.74 | 25.89 |
| S2-3 | 7 | 28.29 | 28.45 | 32.26 | 28.92 | 20.62 | 21.66 | 28.12 | 29.28 | 26.75 | 26.47 |
|  | 8 | 28.47 | 28.28 | 31.70 | 28.80 | 20.68 | 21.89 | 28.38 | 28.61 | 26.90 | 26.56 |
|  | 9 | 28.53 | 28.04 | 31.76 | 28.96 | 20.43 | 21.27 | 27.87 | 29.46 | 26.78 | 26.47 |
| S3-1 | 1 | 28.02 | 27.97 | 32.49 | 28.67 | 21.59 | 21.77 | 29.16 | 29.03 | 27.48 | 26.03 |
|  | 2 | 27.98 | 27.91 | 32.02 | 28.64 | 21.26 | 21.64 | 29.12 | 29.03 | 27.38 | 26.06 |
|  | 3 | 28.04 | 27.92 | 32.37 | 28.61 | 21.13 | 22.14 | 28.76 | 29.44 | 27.26 | 26.16 |
| S3-2 | 4 | 28.13 | 28.03 | 32.65 | 28.23 | 21.19 | 21.66 | 28.36 | 29.41 | 27.18 | 26.49 |
|  | 5 | 28.08 | 27.99 | 32.00 | 28.14 | 21.08 | 21.97 | 28.33 | 29.17 | 27.16 | 26.31 |
|  | 6 | 27.99 | 27.90 | 32.27 | 28.36 | 20.89 | 21.85 | 28.18 | 29.24 | 27.23 | 26.49 |
| S3-3 | 7 | 27.34 | 27.26 | 31.53 | 28.03 | 20.29 | 21.61 | 28.82 | 28.89 | 26.95 | 25.69 |
|  | 8 | 27.37 | 27.29 | 32.06 | 28.04 | 20.40 | 21.43 | 28.90 | 28.96 | 27.01 | 25.80 |
|  | 9 | 27.24 | 27.15 | 31.87 | 28.04 | 20.37 | 21.50 | 28.52 | 29.18 | 26.99 | 25.80 |
| S4-1 | 1 | 29.87 | 29.97 | 34.62 | 27.05 | 25.46 | 23.30 | 28.61 | 31.49 | 26.77 | 28.08 |
|  | 2 | 30.03 | 29.88 | 34.76 | 26.97 | 25.77 | 22.98 | 28.79 | 30.73 | 26.94 | 28.05 |
|  | 3 | 30.01 | 29.88 | 34.04 | 27.24 | 25.81 | 23.15 | 28.83 | 31.25 | 27.01 | 28.28 |
| S4-2 | 4 | 27.44 | 27.95 | 31.76 | 25.16 | 23.59 | 21.80 | 26.78 | 28.92 | 25.44 | 25.45 |
|  | 5 | 27.42 | 27.70 | 32.75 | 25.14 | 23.94 | 21.64 | 26.78 | 28.73 | 25.40 | 25.26 |
|  | 6 | 27.50 | 27.54 | 31.82 | 25.20 | 24.36 | 21.98 | 27.02 | 29.45 | 25.45 | 25.29 |
| S4-3 | 7 | 27.09 | 27.24 | 31.09 | 25.12 | 23.38 | 21.73 | 26.53 | 28.11 | 25.67 | 24.64 |
|  | 8 | 27.12 | 27.26 | 31.10 | 25.04 | 23.76 | 21.59 | 26.53 | 28.42 | 25.65 | 24.92 |
|  | 9 | 27.09 | 27.25 | 31.16 | 25.02 | 23.14 | 21.51 | 26.45 | 28.34 | 25.69 | 24.81 |
| S5-1 | 1 | 28.37 | 27.51 | 32.23 | 25.81 | 21.59 | 21.86 | 28.03 | 29.45 | 26.69 | 26.01 |
|  | 2 | 28.40 | 27.52 | 32.38 | 25.73 | 21.53 | 22.22 | 27.92 | 29.55 | 26.79 | 26.46 |
|  | 3 | 28.34 | 27.56 | 32.31 | 25.68 | 21.64 | 22.03 | 27.99 | 29.70 | 27.02 | 26.11 |
| S5-2 | 4 | 28.33 | 27.30 | 31.88 | 25.40 | 20.67 | 21.39 | 27.11 | 28.22 | 26.37 | 26.15 |
|  | 5 | 28.17 | 27.29 | 32.46 | 25.37 | 20.75 | 21.31 | 26.52 | 28.78 | 26.44 | 26.12 |
|  | 6 | 28.26 | 27.35 | 32.04 | 25.52 | 20.37 | 21.59 | 26.81 | 28.86 | 26.35 | 25.99 |
| S5-3 | 7 | 29.29 | 28.31 | 33.34 | 26.24 | 22.12 | 22.23 | 28.30 | 30.06 | 27.11 | 27.26 |
|  | 8 | 29.22 | 28.51 | 33.30 | 26.32 | 21.67 | 22.39 | 28.10 | 30.35 | 27.06 | 27.16 |
|  | 9 | 29.37 | 28.30 | 33.32 | 26.37 | 22.00 | 22.45 | 28.11 | 30.48 | 27.02 | 27.14 |
| S6-1 | 1 | 32.67 | 32.36 | 36.37 | 29.03 | 26.47 | 24.35 | 33.40 | 33.20 | 29.17 | 30.91 |
|  | 2 | 31.47 | 32.29 | 35.04 | 28.92 | 26.41 | 24.39 | 32.88 | 32.10 | 29.22 | 30.96 |
|  | 3 | 30.14 | 32.16 | 34.47 | 29.08 | 26.54 | 24.28 | 33.03 | 33.56 | 28.90 | 30.81 |
| S6-2 | 4 | 29.42 | 29.90 | 33.56 | 26.67 | 24.02 | 22.98 | 30.82 | 31.01 | 28.04 | 27.63 |
|  | 5 | 29.85 | 29.51 | 33.38 | 26.71 | 24.31 | 22.72 | 30.91 | 31.26 | 27.92 | 27.61 |
|  | 6 | 29.58 | 29.78 | 33.21 | 26.65 | 24.17 | 22.93 | 31.18 | 30.85 | 27.95 | 27.54 |
| S6-3 | 7 | 30.08 | 29.80 | 33.45 | 26.65 | 24.29 | 22.67 | 31.37 | 30.74 | 28.09 | 28.65 |
|  | 8 | 30.22 | 30.21 | 34.25 | 26.85 | 24.01 | 22.48 | 30.28 | 31.01 | 28.02 | 28.52 |
|  | 9 | 30.30 | 30.17 | 33.38 | 26.79 | 24.29 | 22.71 | 31.02 | 31.05 | 28.16 | 28.26 |
| S7-1 | 1 | 27.83 | 25.57 | 29.16 | 24.26 | 22.92 | 20.26 | 24.66 | 27.52 | 25.63 | 23.33 |
|  | 2 | 26.29 | 25.64 | 28.88 | 24.10 | 22.55 | 20.62 | 24.62 | 27.50 | 25.34 | 23.47 |
|  | 3 | 26.41 | 25.55 | 29.01 | 24.20 | 23.02 | 20.41 | 24.62 | 27.63 | 25.63 | 23.46 |
| S7-2 | 4 | 28.32 | 26.71 | 32.73 | 24.96 | 24.31 | 21.23 | 26.24 | 30.11 | 26.43 | 26.09 |
|  | 5 | 28.03 | 26.90 | 32.43 | 24.82 | 23.95 | 21.25 | 26.17 | 29.89 | 26.76 | 26.00 |
|  | 6 | 27.85 | 26.91 | 32.09 | 25.12 | 24.18 | 21.41 | 25.93 | 29.76 | 27.08 | 26.23 |
| S7-3 | 7 | 26.48 | 25.33 | 29.54 | 24.01 | 22.94 | 20.79 | 24.74 | 27.65 | 25.22 | 23.47 |
|  | 8 | 26.15 | 25.38 | 29.45 | 23.86 | 22.57 | 20.49 | 25.02 | 28.00 | 25.11 | 23.33 |
|  | 9 | 26.30 | 25.29 | 29.88 | 23.76 | 23.51 | 20.66 | 24.59 | 28.10 | 25.61 | 24.01 |
